# A Lasting Impression: Exploring the Meaningfulness of a Singular Moment

**DOI:** 10.1177/10497323231201254

**Published:** 2023-11-27

**Authors:** Michael van Manen

**Affiliations:** 1 Department of Pediatrics, 3158 University of Alberta, Edmonton, AB, Canada; 2 John Dossetor Health Ethics Centre, 3158 University of Alberta, Edmonton, AB, Canada

**Keywords:** phenomenology, lived experiences, ethics, moral perspectives, bereavement, grief

## Abstract

How do we explore the meaning and meaningfulness of a singular event that lives on with us as a lasting impression? What are the initial beginnings and final endings of such living moments? How do we make sense of the significance of events that are so meaningful that they have become a lasting impression. This paper focuses on the phenomenology of such lasting impressions, by drawing on an exemplary anecdote about parental bereavement in newborn intensive care. The phenomenological intent is to determine the depth and magnitude of moments that as healthcare providers we may all too easily miss. As well, the methodological intent is to show how as researchers we can engage in a qualitative manner with empirically obtained experiential material.

Allow me to begin by sharing an anecdote from a newborn intensive care unit (NICU) in western Canada. It consists of the words of a parent whose child died following a decision to discontinue mechanical ventilation. He passed away peacefully, held and loved by his family. But then, in a reflective moment, the mother recounts what it was like to leave her son after his death.

“There’s one thing that I regret. And that’s when I was leaving, I just took one more glance into the room. And it’s a very weird being and seeing situation, where for the past three weeks, your son is in a room and he’s got these tubes, he’s got these monitors, he’s got everything and everyone that’s looking after him. There’s always somebody in the room watching him. And then when you leave, he’s the only one in the room. There’s no monitors on, he’s sleeping in his bed, and that’s all you see. And that’s one memory that I wish I didn’t have to experience. It’s really painful to think back to.”^1^

Some may say that a memory is simply a lasting impression—the physiology of which corresponds to the organization of cortical neurons as a consequence of sustained activation (Zacks, 2020).^2^ A memory can be understood to consist of the experiential residue, the remains, the remnants of a past that we recall in our present. And since it is the past, we should be able to deal with and disregard our memories: over and done, as bygone events. But, of course, this is not true. We live our memories: our past in the present. And some events have so deeply impressed on us, it is as if they have “scarred” our very cerebrum (James, 1918, p. 670).

Can we speak of a lasting impression as a phenomenon? As a full-fledged distinguishable experience? What causes a particular impression to remain? Why this one rather than that one? Can we suppress or alter such memories? At what point are they pathological? As we wonder about these questions, we may recognize a tendency to think about a lasting impression in a causal manner. After all, in our day-to-day lives we are constantly encountering people, places, and things that may or may not be constitutive of memories. Some days pass by that seemingly leave us untouched. Perhaps such days are empty or relatively routine. Nothing particularly interesting happened. But there are also moments, however brief they may have been, that have pressed hard on us, and perhaps we have pressed hard on them. Some of these are joyful moments, the birth of a child; humorous moments, the delivery of just the right joke; traumatic moments, being subjected to violence; prideful moments, a celebrated accomplishment; we could go on. These impressions, of course, all vary in their texture, intensity, and vividness. Some moments, we may like to return to, or revisit in our imagination. We think of them fondly as we fall asleep. Yet, other moments may be intrusive, haunting us, disturbing our routines.

What is the lived meaning of a lasting impression? What are the experiential beginnings and endings of such impressions? How do we understand the meaning of memories as lasting impressions? Is an impression the same as a memory? Or are impressions less firm, less definite, more unsure, vague, unclear, more easily mistaken or remembered wrongly, or stuck with emotional confusions? Some experiences are so intense and overwhelming that they disappear as amnestic syndromes: strangely, they cannot be remembered at all. Others are so powerful that we cannot let go of their obsessive presence as we are constantly reminded of them and must relive them.

When do we call a moment impressive? What can leave a lasting impression? What kind of remembrance is impressively impressional? The concern is neither simply with their physiology nor their psychology. Instead, we ask these questions from a wondering about their phenomenality: the living meanings that have impressed on us so deeply that we forever live them and live with them. Still, not all last impressions are lasting. Some impressions elude our attempts to recall them in their impressiveness. It is as if what persists is vague, equivocal, or poorly formed.

I want to focus on the meaningfulness of those singular moments that press upon us with the imprints of lasting impressions. They visit us again and again and again. We may have very specific emotions associated with them—regret, despair, joy, happiness, fear, anger, love—or they may be somewhat undefined in their surreality. And we may also acknowledge that some impressions have a dormant latency whereby their meaning is only later and gradually revealed. The latency of such impressions is their meaningfulness, a meaning that is not merely significant because it lasts but also because it grows in depth. Such meaningfulness is not just from the past but rather from how we recall memories in our present. In the above anecdote, the mother expresses regret about the final glance she took at the room and her dead child in the room. We wonder about the meaningfulness of this lasting impression, the experience of the final moment that the mother shares, by recalling her words:

## “There’s One Thing That I Regret. And That’s When I Was Leaving, I Just Took One More Glance Into The Room …”

The anecdote opens with an important series of phrases that deserve our attention. We may consider their meaning based on both what was said and what was left unspoken, but that nonetheless make such words possible to be uttered. “There’s one thing that I regret”: a lasting impression may show itself among others things, to be felt in its recollection, as meaningful. “And that’s when I was leaving”: a lasting impression has its own particular moment, its own particular temporality. “I just took one more glance into the room”: the phenomenon is disclosed in a moment, for the last time. These preliminary reflections reveal a truth. If the mother had not stayed with her child for a meaningful period—a time of many glances, gazes, and contemplations—then the mother would not and could not have taken “one more glance” for a last time. Yet, enigmatically, it is the “glance” that produced the “impression,” a glance that she could not help but take.

It seems to be a contradiction that what is formative of a lasting impression, “one more glance,” may be so transitory and spontaneous in its occurrence. The literature on memory formation tells us that we are more assured to remember, to commit to so-called long-term memory, if the provocation is especially remarkably unusual or of a longer duration as a consequence of neural substrates (Jordão & St. Jacques, 2022; Merz & Wolf, 2022; Williams et al., 2022). But what is more fleeting than a glance? It is the briefest of looks. Barely a second or it would be more than a glance; it would be something else. Would a careful or meditative look have produced something else?

We may reflect on the usual nature of our engagement in the world which elapses into memory whereby we pass through life, completing various activities, perform particular tasks to achieve some ends. We normally cannot remember all of our glances, gazes, scans, and looks that composed a day, just as we cannot necessarily recount in vividness all of our other day-to-day interactions with family, friends, or colleagues. As Edward Casey (2000) says,

Certain human activities are essentially remainderless. They merely take place—and exhaust themselves in their occurrence without leaving any significant residue … [they] do not precipitate themselves into our subsequent life in any lasting way. (p. 266)

Impressions tend to be short, pressing, and momentary pushes. From a stamp, imprint, or other pressing means, their residues are marks and markings. And yet, the enigmatic thing about a lasting impression is that despite its possibly ephemeral nature, it may fail to fade with the passing of time. It stays with us after it has happened. It stands out as that “one thing” from the stream of our consciousness as something primal that has affected us, leaving us changed by its residue. And so, we may have regrets, remorse, or unresolved emotions tied to such events.

The word “impression” is from the Old French *impression* as “a pressing on the mind” and from Latin *impressionem* as “a pressing into, onset, attack.” There is a force to an impression, a literal “pressing into” from *imprimere*. To be pressed upon is to be touched, even if we are touched purely through the contact of the eyes, a glance. The impression has power, to be sure. It is a physical and aesthetic force that leaves us changed. Impressionism was an artistic style that used the play of light and darkness to create visual impressions. The first aesthetic use of the term impressionism derives from the painting by Claude Monet, entitled *Impression*, *Sunrise*, 1873. It depicts the sparkling image of sky and water surrounding a small dark boat in the seaport of Le Havre. The tiny vessel is seen as if through an expressive emotional haze of streaks of tears. It is indeed an expressive impression of an existential temporal rising morning sun.

And so, we may begin by reflecting that the phenomenality of a lasting impression far exceeds its initial duration. With a glance, a parent may see what cannot be unseen. Its brevity does not determine and constrain its significance. Perception has pressed onto the mother of the deceased child. How could she not have looked back? To take one more look.

## “… And It’s a Very Weird Being and Seeing Situation …”

Sometimes we may wonder about how people describe their experiences. Are their words quite right? Are “weird being” and “seeing situation” illuminative of meaning? Do these phrases aptly point to the eidetic (singular) meaning of a lasting impression? How do these loculations relate to the experience as it was lived through in the moment of its occurrence, the past, compared to how it is recalled in its presence or in some distant future? The answers to such questions are elusive, and yet from these words of “a very weird being and seeing situation,” we may consider the experiential autonomy of such lasting impressions.

It is quite possible that the woman’s talking about that last moment when she walked out of the room where her baby’s body was now lying unattended in its hospital crib might have been somewhat differently described if she had written down the recent memory of that moment. Would she have used the word “glanced” to describe this last look in writing?

As its own moment, a particular “being” and “situation,” we may recognize that at times we find ourselves in a lasting impression as it turns from our unreflective consciousness to reflective awareness. This is not to say that a lasting impression is dissociated from the flow of time; rather, we may recognize that those most pointed of lasting memories are conspicuous. Like the punctum of a photograph, the image is of a form that reflects the world as it was lived in that moment. Much like a picture that we may look at in the manner of Roland Barthes (1980/1982; 1984/1986), as studium (for the sake of interest) or as punctum (because it expresses a fundamental meaning), we find ourselves in the “being” of the “situation.” While we may not recall what happened directly before or after such a moment, as if the temporal borders of the memory are unclear, the memory itself has its own resolution as an impression. William James (1918) writes:

The stream of thought flows on; but most of its segments fall into the bottomless abyss of oblivion. Of some, no memory survives the instant of their passage. Of others, it is confined to a few moments, hours, or days. Others, again, leave vestiges which are indestructible, and by means of which they may be recalled as long as life endures. (p. 643)

Consider how we may speak commonly about first impressions: those almost instant conclusions we draw when we first meet someone or experience something for the first time as a “first impression.” And we know first impressions matter to the extent that we value them: was it a good or bad impression? The lay literature is full of advice on the do’s and don’ts of first impressions: make eye contact, avoid interrupting, be authentic, and so forth. While there can only be one first and one last impression, there is a singularity to the last impression that transcends the first. Here is the insight: A first impression is evanescent in the wake of a last impression. If we make a poor first impression, we can make amends, apologize, or otherwise improve upon it. The last impression, however, has finality, and it is the “last one.” It is the impression, if it lasts, that we are left with, as a forever impression.

In this way, the last lasting impression may stand out from the stream of consciousness of our memory in its exceptionality. There is an extraordinariness to the moment, even if it occurs in an everyday moment of ordinariness. And in the moment of a lasting impression, we may both be in that moment yet also experience that moment as a recollection as we lived through it. The lasting last impression lacks a true antonym.

## “… where for the Past Three Weeks, Your Son is in a Room and He’s Got These Tubes, He’s Got These Monitors, He’s Got Everything and Everyone That’s Looking After Him. There’s Always Somebody in the Room Watching Him …”

These few lines express the experiential concreteness of these memories. A lasting impression is not abstract. It is not an idea or schema. We do not need to memorize last impressions. And we cannot be simply told of an event and experience it as a lasting impression, as if it is our own. While psychologically we may wonder at what depths of our psyche such impressions are formed, phenomenologically we know it is through our direct experiencing of the world that our perceptions become impressions. And yet, to experience an impression, we may wonder if one needs to be impressionable.

Now, what could be more impressionable than the parent of a child who has required care in a NICU? Such parents have lived through the exultation of a new beginning, a new life being born, only to be faced with the medical reality of prematurity, congenital anomalies, or some other clinical complications necessitating intensive care: “tubes,” “monitors,” “everything and everyone.” Even the hardest of souls softens under the wear of such experiences. And, in the techno-medical context of a NICU, abundant with medical technologies and medical staff, parents hopefully come to know this place as a place where their child is looked after, cared for, and cared about (van Manen, 2019).

Care is in and of itself meaningful. And to reflect on the meaningfulness of care as a topic deserves sustained attention (van Manen, 2002). “Ethics permeates the stories of each and every child receiving medical care, even the so-called uncomplicated cases, manifesting something originary that touches us” (van Manen, 2021, p. 9). In moments of care—closeness, intimacy, and responsibility—we may hear the words of Cicero (1855), “the life of the dead is placed in the memory of the living” (p. 138). This is significant for the meaningfulness of a lasting impression. This memory is not conceived from the death of a child but instead from their having lived. Following, a lasting impression is more than a simple detail or other understanding known to be factually true. It is not just that the parent recalls a memory of their child as an occurrence. Rather, in the lasting impression, they recall their perception as it was conceived in their lived experience of their child living in the NICU. This is not quite right, but we need to say it as such for now.

## “… And Then When You Leave, He’s the Only One in the Room. There’s no Monitors on, He’s Sleeping in His Bed, and That’s All You See …”

While we may be tempted to consider the mother as leaving her son’s body after his death, the mother does not speak of (mention) his bodily remains. Instead, she leaves “him” as “the only one in the room,” still “sleeping in his bed” as if he was still alive. We may even wonder whether phenomenologically her son was really dead (in the sense of a dead body) in this final moment and, therefore, whether she always will be burdened with the impression that she left him in that lonely room: a room now tainted with this last and lasting impression.

As readers, we may hear the reverberations—“that’s all you see”—of such a lasting impression that will endure in the life of a parent. And yet, we should also acknowledge that the mother’s words while expressing truth do not speak it. The truth is that the mother sees so much more than she says or speaks of. She sees so much more than the physicality of her child lying there, in his bed, now without monitors, alone in the room. Phenomenologically, what she sees is her child’s vulnerability and abandonment, even in his death. This is a tentative yet necessary wondering reflection that we must engage.

From three weeks of hospitalization, the lasting impression assuages and eclipses what came before it, all of the care he received. It is not that the moment wholly negates the time and care that he received in the NICU. It is not an experiential erasure of the past in the present. But it may unsettle a parent’s previous perceptions, haunting their conscience. And if such perturbations lead the parent to question the care that their child received from them or the hospital staff, we can appreciate just how profoundly upsetting such a lasting last impression may be. In this way, the moment of a lasting impression implicates all of us. We recognize our activity or passivity as moral action or inaction. In such moments, it is in the fullest normative sense that our actions are valued as right or wrong, good or bad. By definition, impressive experiences do not leave us ethically untouched.

## “… And that’s One Memory That I Wish I Didn’t Have to Experience. It’s Really Painful to Think Back to.”

If we accept that we live our memories beyond their past, it would be a mistake to pass over the duration of a last impression. Said differently, we would be misguided to reduce the significance of a lasting last impression to some form of fixed interval or period of time. It is quite the opposite: the lasting impression transcends time in its persisting presence in our lives. In *On Time and Being*, Heidegger (1969/2002) writes:

To presence means to last. But we are too quickly content to conceive lasting as mere duration, and to conceive duration in terms of the customary representation of time as a span of time from one now to a subsequent now. To talk presencing, however, requires that we perceive biding and abiding in lasting as lasting in present being. What is present concerns us, the present, that is: what, lasting, comes toward us, us human beings. (p. 12)

What lasts has a lasting presence, and perhaps will never leave us. And what is more, what lasts comes into presence is its present being, meaning in those moments of remembrance. Consequently, we should understand that while we may recognize the significant latency of a lasting impression in its moment of perception, other meaning may only come to our awareness later in our lives. Just consider those moments that may have seemed of little significance in our childhood or youth that in our later life are realized in their significance. The phenomenological insight here is that there are some moments of our lives that we never quite finish living through. These memories do not dwell solely in the past at all but instead live in the present as we may relive them, to continue to live through them, in our present lives.

Can we speak to a good or bad lasting impression? Are painful impressions bad and comforting impressions good? Is morality dichotomous when it comes to our lasting impressions? Perhaps because of their lasting, their inability to exhaust themselves, and their incompleteness, we need to acknowledge that through the flow of time normative aspects of experiences may be revisited and reinterpreted. In other words, it is possible that what was painful in the past becomes a source of comfort in the future. Or that those memories that haunt us are also memories that we will treasure. Still, we should also appreciate that there are aspects to lasting impressions that are fixed, set, or otherwise have permanence. For our memories, we cannot simply change, distort, or otherwise try to imagine another narrative. If we try to imagine an alternative past—for example, instead of walking away we imagine holding our child one more time, or perhaps our fateful glance instead revealed a nurse walking into the room to stand by our child, or some other happening—we realize that our past memory cannot be revised, substituted, or otherwise unfolded into that which we can more comfortably recall. In this way too, our last impression is lasting.

## Closing Reflections

When we hear the words of our patients and/or family members, we may realize the significances of those most ordinary and extraordinary moments in their lives. Their words may touch our very sensibilities in an ethical reflective manner, during which we may be tempted to draw personal, psychological, or other meaning interpretations into the processes of their individual sense-making. However, when weighing the meanings of the words that arise in the reading of spoken texts, we must be cautious of any act of de-construction. Ricoeur (1978) says, “The text is a complex entity of discourse whose characteristics do not reduce to those of the unit of discourse, or the sentence” (p. 259). There are often multiple meanings revealed by a text, both implicit and explicit, in addition to ambiguities in meaning.

When it comes to psychological reflection, we need to consider how our reflecting on expressions of someone’s lived experience is unavoidably personal and, potentially, removed from the finalities of phenomenological meaning as it arises in those moments we live through. For this article, I have refrained from describing how this text was developed methodologically. There exist various sources that explore possibilities for phenomenological research and writing (see van Manen, 2023). However, I would like to emphasize that at the heart of this kind of writing is the appreciation of the need for phenomenological work to maintain an attitude of tentative wondering on the meaningfulness of concrete experiential examples such as the mother’s anecdote of the final glance provided at the opening of this text (see van Manen & van Manen, 2021).

The practice of health ethics frequently focuses on matters of “opening ethics” such as obtaining informed consent, building rapport, and other issues related to autonomy, shared decision-making, and so forth. In comparison, the term “closing ethics” points to how we bring our relationships with others to a close or otherwise how we conclude our involvement in their care. When we read the opening anecdote again, as healthcare professionals, we can sense empathically that this moment would be painful to remember. Something different could have been done and probably should have been done in the final moments of care that this family received. We should have attended to our closing ethics. Parents live these final moments that transcend the passage of time, that perhaps are never truly in the past. As Hermann Broch (1987/1989) wrote, “No one’s death comes to pass without making some impression, and those close to the deceased inherit part of the liberated soul and become richer in their humanness” (p. 22).

As healthcare providers support parents at the end of their child’s life, we need to keep in the forefront of our minds that final moments are heavy with meaning. Even if some moments pass as fleetingly as a glance, we need to understand how parents are caught in the latency of those moments that have consequences for living the “loss” of their child well beyond their time in hospital, for the meaningful remainder of their lives.

## Notes

1. The opening anecdote in this paper comes from the article “Exploring and Supporting Parents Stories of Loss in the NICU: A Narrative Study” authored by Jenna Lakhani, Cheryl Mack, Dianne Kunyk, and myself (2023). I am gracious to these colleagues for this work, as well as the families that participated in this study who openly shared their meaningful experiences and valuable perspectives.

2. Theorizing on memory formation, distinctions may be made between sensory registers, short-term memory, and long-term memory (Camina & Güell, 2017). And, further divisions can be drawn between those long-term memories that can be explicitly recalled (episodic and semantic) compared to those which resist explication (procedural, associative, non-associative, and priming). Categorically, a lasting impression may be made into an object for scientific inquiry to be studied as episodic memory. For example, we can research episodic memories as neural representations, recognizable patterns of brain states that reflect the previously experienced episodes (Heinen et al., 2023). This kind of focus, however, passes over experiential aspects of memory. The study of memory is not only the sole purview of the natural sciences; interdisciplinary contributions have also been made by those in the health sciences and humanities (Heux et al., 2023).
